# P-969. Antibiotic Prescribing Patterns for Surgical Prophylaxis Before and After an Educational Campaign about Penicillin Allergies

**DOI:** 10.1093/ofid/ofae631.1159

**Published:** 2025-01-29

**Authors:** Megan Backus, Marianne Romanos, Anthony Wasielewski, Natalie Paz, Timothy Gauthier

**Affiliations:** Baptist Health Boca Raton Regional Hospital, Gainesville, Florida; Baptist Health Boca Raton Regional Hospital, Gainesville, Florida; Baptist Health Boca Raton Regional Hospital, Gainesville, Florida; Baptist Health Boca Raton Regional Hospital, Gainesville, Florida; Baptist Health South Florida, Miami, Florida

## Abstract

**Background:**

Patients with a documented penicillin allergy often do not receive first-line antibiotics for surgical prophylaxis due to the concern for allergic cross-reactivity between cefazolin and penicillins. Receiving suboptimal antibiotics unnecessarily may contribute to increased risk of adverse drug reactions and undesirable clinical outcomes. This study evaluated the rate of first-line antibiotic regimen utilization for surgical prophylaxis in penicillin allergy-labeled patients before and after a prescriber-facing educational campaign about penicillin allergies.

**Figure 1.**
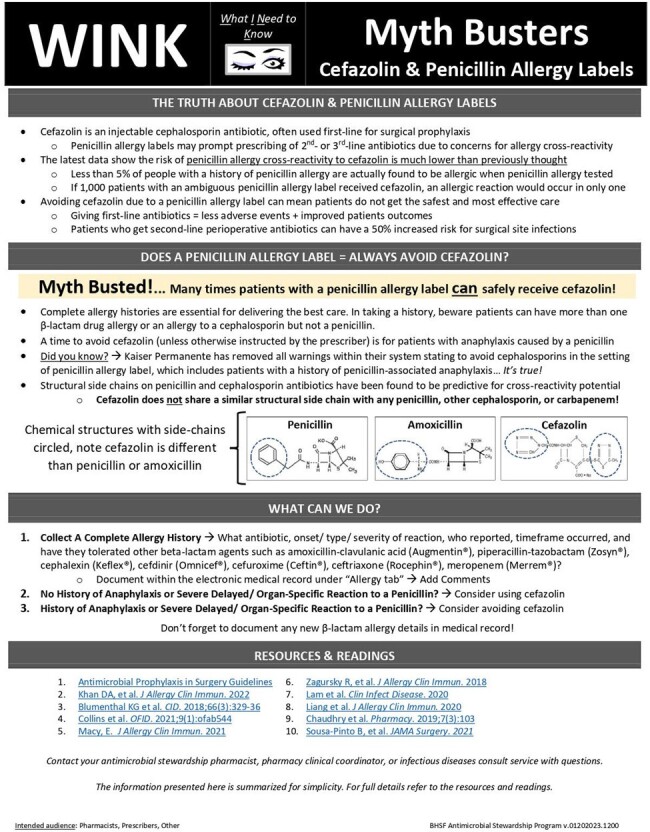
WINK Educational Document

**Methods:**

This was a single-center retrospective cohort study conducted at a tertiary care community hospital. Education was provided over one month in-person by infectious diseases and surgical services pharmacists to surgeons and anesthesiologists, including review of an educational handout detailing current penicillin allergy label literature, cefazolin side-chain dissimilarity, and when cefazolin should be considered or avoided (Figure 1). This content was also provided to pharmacists and posted to the system intranet. Patients were included if they had a documented penicillin allergy and received an antibiotic during admission for procedural prophylaxis. Patients receiving systemic antibiotics or with an infection within three days of procedure were excluded. The primary outcome was rate of first-line pre-operative antibiotic regimen utilization for surgical prophylaxis pre-education versus post-education. Secondary outcomes included all-cause hospital readmission within 90 days, acute care length of stay, surgical site infection rate, and safety outcomes. This study was approved by the local Institutional Review Board.

**Table 1.**
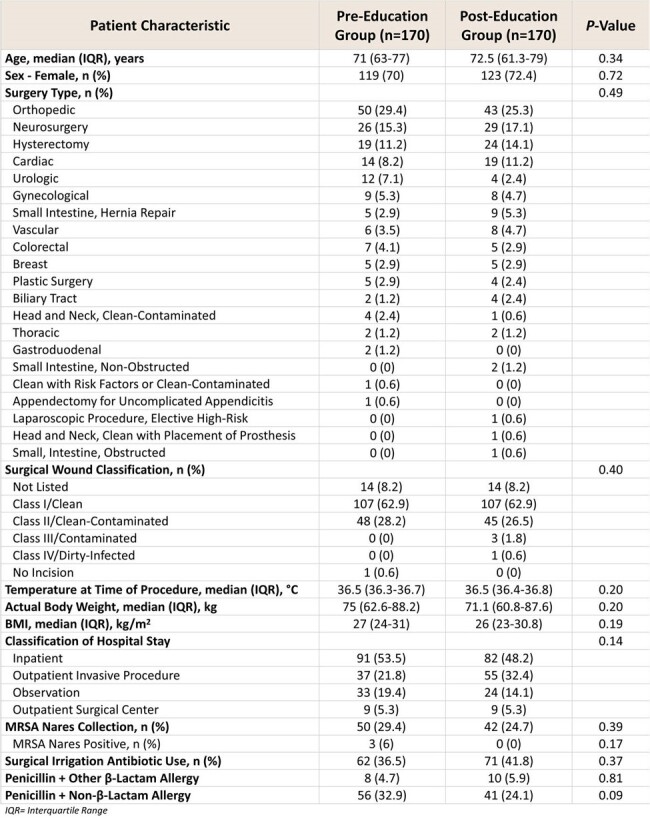
Baseline Patient Characteristics

**Results:**

340 patients were included in the study, evenly split into the pre- and post-groups. Patient demographics are shown in Table 1. First-line pre-operative antibiotic regimen utilization occurred in 89 patients in the pre-education group (52.4%), and in 113 patients in the post-education group (66.5%) (P = 0.011). Secondary outcomes are listed in Table 2.

**Table 2.**
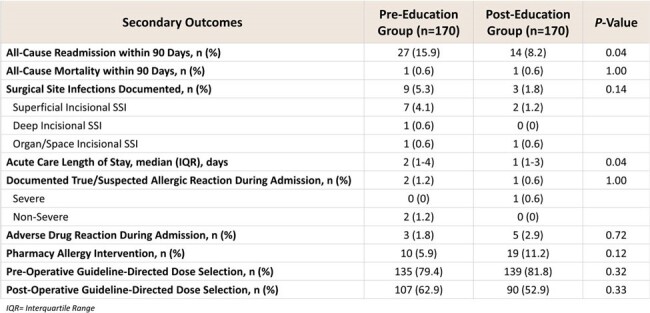
Results for Secondary Outcomes

**Conclusion:**

An educational campaign focusing on reasonable use of cefazolin in the setting of a penicillin allergy label may improve use of first-line antibiotics, which may positively influence clinical outcomes.

**Disclosures:**

**Timothy Gauthier, PharmD, BCPS, BCIDP**, AbbVie Pharma: Advisor/Consultant|Antimicrobial Therapy, Inc: Advisor/Consultant|Ferring Pharma: Advisor/Consultant|Firstline Mobile Health: Advisor/Consultant|Gilead Pharma: Advisor/Consultant|GoodRx: Advisor/Consultant|GSK Pharma: Advisor/Consultant|Melinta Pharma: Advisor/Consultant|Pattern Biosciences: Advisor/Consultant|Pfizer Pharma: Advisor/Consultant|ProCE: Honoraria|WWW.LearnAntibiotics.com: Ownership Interest

